# Functional outcome of 103 fractures of the proximal fifth metatarsal bone

**DOI:** 10.1186/s40001-021-00623-6

**Published:** 2021-12-20

**Authors:** Patrick Pflüger, Michael Zyskowski, Michael Müller, Chlodwig Kirchhoff, Peter Biberthaler, Moritz Crönlein

**Affiliations:** Department of Trauma Surgery, Klinikum rechts der Isar, Technical University of Munich, Ismaninger Str. 22, 81675 Munich, Germany

**Keywords:** Fifth metatarsal fracture, Lawrence and Botte, Treatment outcome, Follow-up study

## Abstract

**Background:**

Metatarsal fractures are common skeletal injuries of the lower extremity in adults. The majority involves the proximal fifth metatarsal bone. In the current literature, there still exists controversy regarding treatment recommendations for the different fracture entities.

**Methods:**

All patients suffering from single fractures to the proximal fifth metatarsal bone between 2003 and 2015 were enrolled in this retrospective analysis. Only patients with a minimum follow-up of 12 months were included. The fractures were classified according to Lawrence and Botte (L&B). Data were collected via patient registry, radiographs and a standardized questionnaire (Foot and Ankle Outcome Score = FOAS). For outcome analysis, the nonparametric Mann–Whitney *U* test was performed and Spearman’s rank correlation coefficient calculated.

**Results:**

In total, the functional outcomes of 103 patients suffering from fractures to the proximal fifth metatarsal bone were analyzed. L&B type I fractures (*n* = 13) had a FAOS score of 91 ± 23, L&B type II (*n* = 67) presented a score of 91 ± 15 and L&B type III (*n* = 23) a score of 93 ± 11. Surgically treated patients with an L&B type II fracture had no statistically significant better functional outcome in comparison to conservative management (*p* = 0.89). Operatively treated L&B type III fractures tended to have a better functional score (*p* = 0.16). The follow-up time was 58 (min: 15; max: 164) months.

**Conclusions:**

Overall, the functional outcome following fractures to the proximal fifth metatarsal bone is satisfactory. Conservatively treated L&B type II fractures showed an equivalent functional outcome compared to surgical management. Patients with an L&B type III fracture mainly were treated surgically, but difference in FAOS score did not reach level of significance.

## Background

Metatarsal fractures are one of the most common injuries of the midfoot with an incidence of up to 75 persons per 100,000 per year among adults [1–3]. More than half of all metatarsal fractures involve the fifth metatarsal bone and the majority is located at the proximal end [1, 3]. The peak incidence of fifth metatarsal fractures in men is below the age of 40, whereas mostly women older than 50 years are affected [3, 4]. Over time different classification systems were developed according to the location and number of fragments [5–7]. The Lawrence and Botte classification is most frequently used nowadays [8]. The authors distinguish between three types of fractures to the proximal fifth metatarsal bone: type I includes tuberosity avulsion fractures, proximal to the intermetatarsal joint (L&B type I), whereas type II fractures are located at the intermetatarsal joint (L&B type II), and type III fractures are defined as diaphyseal stress fractures, distal to the intermetatarsal joint (L&B type III) [8]. In a rather current review of the literature Polzer et al. concluded that acute fractures to the proximal fifth metatarsal should be classified into ‘epi-metaphyseal’ (beyond the distal end of the intermetatarsal articulation) and metaphyseal fractures (at the distal end of the intermetatarsal articulation) [9]. Their meta-analysis showed, that L&B type I and II fractures have an equivalent outcome and thus can be summarized as ‘epi-metaphyseal’ fractures [9].

In the literature different definitions of ‘Jones fractures’ were developed [10]. Following the classification of Dameron diaphyseal stress fractures, distal to the intermetatarsal joint, are so-called ‘Jones fractures’ (L&B type III) [5]. According to Lawrence and Botte ‘Jones fractures’ are located at the intermetatarsal joint (L&B type II) [8]. Due to the different terminology, there is no standardized treatment recommendation for all fracture types of the proximal fifth metatarsal bone [10]. According to the existing studies L&B type I and II fractures can be satisfactory managed conservatively [11–13]. But there are also studies recommending surgical treatment of ‘Jones fractures’ [14, 15]. L&B type III fractures should be treated surgically due to their higher likelihood of pseudarthrosis following conservative management [10, 16]. Overall the outcome of adequately treated fractures to the proximal fifth metatarsal bone is good to excellent [3].

The aim of the presented study was to provide further evidence for treatment recommendations by analyzing the functional outcome following operative as well as conservative treatment of fractures to the proximal fifth metatarsal bone.

## Methods

After ethical board approval (No: 409/15 S, Technical University of Munich) the retrospective cohort study was conducted between 2003 and 2015 in a level I trauma center. All patients presenting with a fracture to the proximal fifth metatarsal bone at the department of trauma and orthopedic surgery were reviewed for enrollment. Only patients > 18 years, who were capable of giving informed consent, suffering from a closed fracture to the proximal fifth metatarsal bone were enrolled. The outcome of conservatively and operatively managed patients was analyzed. All patients with a fracture at the same leg, multiple metatarsal fractures, pathological fractures, substance abuse, presenting for revision surgery after external operation and with legal guardian were excluded from the study.

For the evaluation of the functional outcome following fracture of the proximal fifth metatarsal bone the Foot and Ankle Outcome Score (FAOS) was used. The FAOS is a self-administered patient-relevant outcome questionnaire consisting of 42 items (range: 0–100). The German version of the FAOS is a valid and reliable instrument for foot and ankle patients [17]. After hospital treatment and standard postoperative visits, patients were invited by mail to complete the FAOS. Only patients with a minimum follow-up of 12 months were included for further analysis. The main focus of the study was the long-term functional outcome following fractures to the fifth metatarsal bone. Unless otherwise stated, i.e., revision surgery or change of treatment, the bone healed uneventfully following operative and conservative treatment.

Fractures to the base of the fifth metatarsal were classified based on radiographs using the Lawrence and Botte classification system [8]. According to Polzer et al. L&B type I and II fractures can be classified as one entity based on the prognosis and therapeutic consequences [9]. Considering this aspect, we performed an additional analysis with these groups.

General data such as age, gender, affected side, date of latest follow-up, time between fracture and surgery and reoperation rate were collected. Operative treatment involved open reduction and internal fixation (ORIF) with screw, plate or K-wire osteosynthesis. The treating senior trauma surgeon specialized in foot and ankle surgery evaluated the type of fracture and determined the treatment. Indications for surgery were comminution, displacement > 2 mm, age and functional demand. The forms of fixation were determined by the treating senior trauma surgeon depending on the type of fracture (localization, comminution, bone quality). Patients with conservative treatment performed partial weight bearing with 15 kg for 6 weeks with crutches and a walking boot undergoing regular radiographic evaluations. After 6 weeks and proper radiographic follow-up patients were allowed to remove the walking boot and start with full weight bearing. The standard outpatient aftercare involves visits to the ambulatory facility with radiographic follow-ups 6 weeks, 3 months and 1 year after trauma.

### Statistics

Data were presented as median ± standard deviation (SD) or minimum and maximum (min; max). RStudio [RStudio Team (2020). RStudio: Integrated Development Environment for R. RStudio, PBC, Boston, MA URL http://www.rstudio.com/] was used for data processing.

Shapiro–Wilk test was performed to test for normality. The nonparametric Mann–Whitney *U* test was used to assess significant differences between two groups. To assess the correlation between two variables, Spearman’s rank correlation coefficient was calculated. *P*-value < 0.05 was considered statistically significant.

## Results

Overall, 116 patients were treated for fractures to the proximal fifth metatarsal bone and 103 patients were available for further data analysis. The median age of the study cohort was 43 ± 19 years, including 63 female (61%) and 40 male (39%) patients. Female patients had an age of 52 ± 20 years and male patients of 39 ± 16 years. The age did not affect the functional outcome (*r* = 0.09, *p* = 0.34). In 53 patients (51%) the right side was injured, whereas 50 (49%) patients fractured their left proximal fifth metatarsal bone. The follow-up time was 58 (15; 164) months (Table 1).

**Table 1:** Overview of general data and functional outcome of patients with fractures to the proximal fifth metatarsal bone classified according to L&B

	Conservative	Operative
Number of patients	60% (*n* = 62)	40% (*n* = 41)
Gender	63% female (*n* = 39)	59% female (*n* = 24)
Side	48% left (*n* = 30)	51% left (*n* = 21)
Age (median, SD, y)	53 ± 20	42 ± 17
L&B I	16% (*n* = 10)	7% (*n* = 3)
L&B II	73% (*n* = 45)	54% (*n* = 22)
L&B III	11% (*n* = 7)	39% (*n* = 16)
Follow-up (median, min; max, months)	55 (15; 122)	68 (16; 164)

### Lawrence and Botte type I

#### General data

There were 13 L&B type I fractures with an age of 59 ± 20 years, including seven female (54%) and six male (46%) patients. In five patients (38%) the right side was injured, whereas eight (62%) patients fractured their left fifth metatarsal bone.

Ten patients were treated conservatively and three patients operatively after seven (4; 28) days. The follow-up time was 37 (22; 150) months.

#### Functional outcome

The median FAOS score was 91 ± 23. Surgically treated patients had a FAOS score of 93 ± 36 and conservatively a score of 88 ± 20.

No patient needed revision surgery and in one of three operatively treated patient hardware removal (K-wires) was performed due to bone consolidation after 4 months.

### Lawrence and Botte type II

#### General data

There were 67 L&B type II fractures with an age of 42 ± 19 years, including 44 female (66%) and 23 male (34%) patients. 33 patients (49%) injured their right side and 34 patients (51%) their left fifth metatarsal bone. 45 patients were treated conservatively and 22 patients operatively after 4 (1; 18) days (Table 1). The follow-up time was 61 (15; 164) months.

#### Functional outcome

**Fig. 1:**
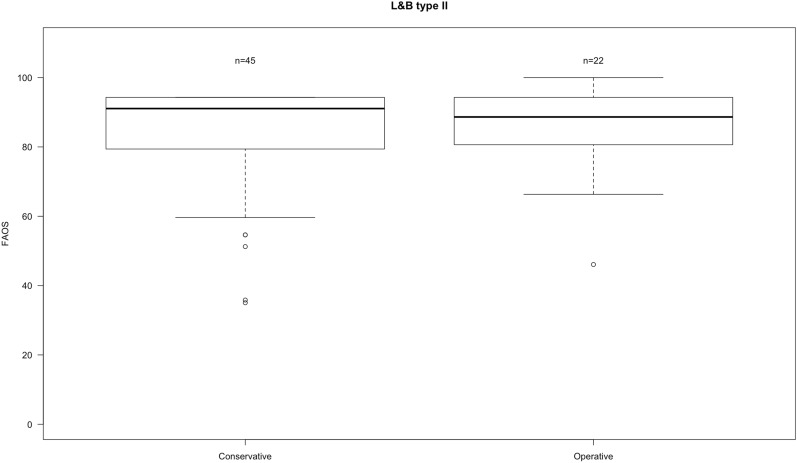
Boxplots of FAOS in L&B type II fractures divided into operative and conservative treatment. Whiskers showing minimum and maximum. *N* number of patients

The median FAOS score was 91 ± 15. Surgically treated patients had a FAOS score of 89 ± 12 and conservatively a score of 91 ± 16 (Fig. 1). There was no statistically significant difference between operatively and conservatively treated L&B type II fractures (*p* = 0.89).

One patient (5%) needed revision surgery due to an infection after 2 months with early hardware removal. Ten patients (45%) had hardware removal due to bone consolidation after nine (4; 19) months.

**Fig. 2:**
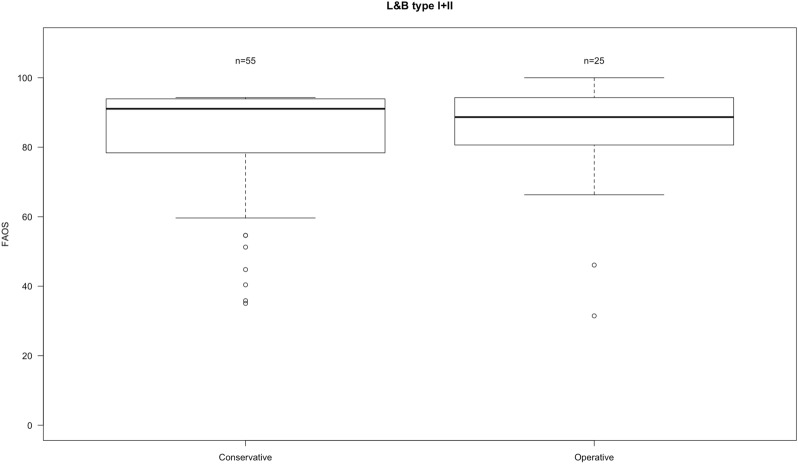
Boxplots of FAOS in L&B type I and II fractures divided into operative and conservative treatment. Whiskers showing minimum and maximum. *N* number of patients

Following the terminology of Polzer et al. the ‘epi-metaphyseal’ group was formed by 80 patients. The median FAOS score was 91 ± 16. Surgically treated patients had a FAOS score of 89 ± 16 and conservatively treated presented a score of 91 ± 16. There was no statistically significant difference between operatively and conservatively treated fractures (*p* = 0.77) (Fig. 2).

### Lawrence and Botte type III

#### General data

There were 23 L&B type III fractures with an age of 45 ± 16 years, including 12 female (52%) and 11 male (48%) patients. In 15 patients (65%) the right side was injured, whereas 8 (35%) patients fractured their left fifth metatarsal bone.

Seven patients were treated conservatively and 16 patients operatively after 4 (1; 12) days (Table 1). The follow-up time was 64 (16; 147) months.

#### Functional outcome

**Fig. 3:**
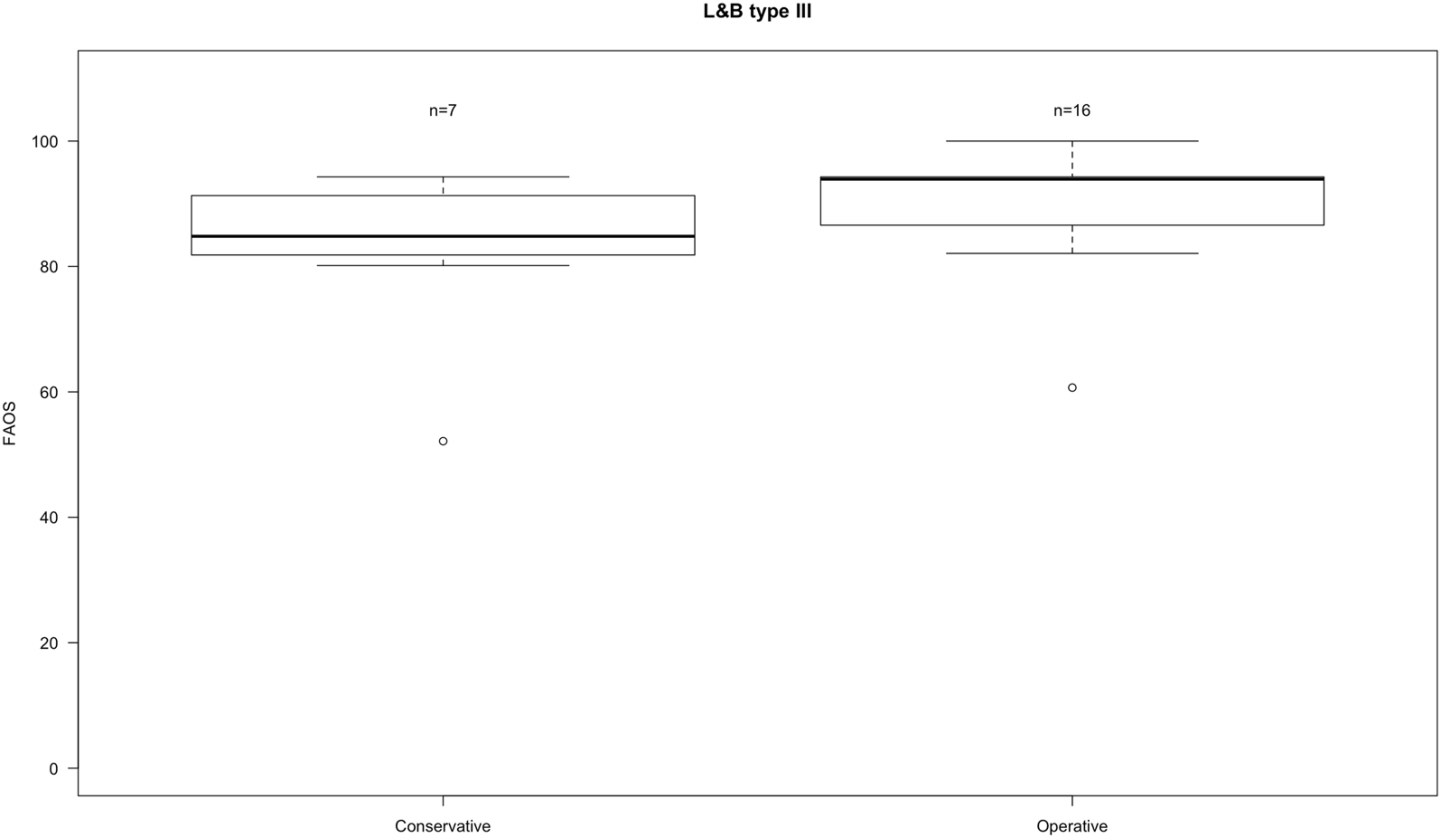
Boxplots of FAOS in L&B type III fractures divided into operative and conservative treatment. Whiskers showing minimum and maximum. *N* number of patients

The median FAOS score was 93 ± 11. Surgically treated patients had a FAOS score of 94 ± 9 and conservatively a score of 85 ± 14 (Fig. 3). Operatively treated L&B type III fractures tended to have a higher FAOS in comparison to conservatively managed patients, but the difference did not reach statistical significance (*p* = 0.16).

Only one patient needed revision surgery due to dislocation and infection after 1 month. Following revision surgery, the bone healed uneventfully and hardware removal was performed 15 months after the initial surgery. In another five patients hardware was removed due to bone consolidation after 12 (11; 16) months.

## Discussion

In general, metatarsal fractures are one of the ten most common skeletal fractures in adults [2]. The majority of these foot injuries affect the proximal fifth metatarsal bone [1, 3]. Treatment recommendations were derived depending on different fracture entities. In the current literature there is still critical discourse whether operative or conservative treatment of fractures to the proximal fifth metatarsal bone is superior [10]. Therefore, the aim of the presented study was to provide further evidence for treatment recommendations by analyzing the functional outcome following operative as well as conservative treatment of fractures to the fifth metatarsal bone.

Overall, the functional outcome of 103 patients suffering from fifth metatarsal bone fractures was assessed. The majority of the patients were female (61%) and on average older than the enrolled male patients. Epidemiological studies of metatarsal fractures also demonstrated a similar age and gender distribution with peak incidences in women around the age or older than 50 years [3, 4].

The functional outcome of L&B type I fractures was satisfactory with a median FAOS score of 91 following a nonoperative treatment (*n* = 10). This is in accordance with other studies advocating a conservative treatment for this fracture type to the proximal fifth metatarsal bone [12, 18, 19]. Outcome was assessed with the Visual Analogue Scale Foot and Ankle Questionnaire (VAS FA) and reported as good to excellent following conservative treatment [19, 20]. The VAS FA is a subjective patient reported score based on 20 questions [21]. In contrast to the FAOS some subscales are underrepresented or missing [17]. The type of conservative treatment included a short walking boot/cast and crutches for 5 weeks or a double-layered elasticated bandage/below the knee-walking cast for 4 weeks [19, 20]. Patients were not advised to partially weight bear and in some cases only received a symptomatic treatment without immobilization. Comparing the 1 year results of Shahid et al. with the presented findings, the long-term outcome after conservative treatment seems to be independent of the treatment protocol [20]. The advantage of a functional treatment with full weight bearing is an early-return-to-work and can be satisfactory employed, independent of displacement, articular involvement and comminution [11, 12].

Patients with an L&B type II fracture presented with a FAOS score of 91 a very good functional outcome. In the analyzed patient cohort conservative management (FAOS = 91) was equivalent in comparison to operative treatment (FAOS = 89) regarding the functional score. Despite some studies supporting surgery in these kind of fractures [14, 15, 22], there is growing evidence in the literature that conservative treatment results in excellent functional outcome [12, 13, 23]. The discrepancies might be due to inconsistent definition of fracture types among the studies since exact fracture location is decisive for prognosis. Following the classification of Dameron diaphyseal stress fractures, distal to the intermetatarsal joint, are so-called ‘Jones fractures’ (L&B type III) [5]. According to Lawrence and Botte ‘Jones fractures’ are located at the intermetatarsal joint (L&B type II) [8]. These inconsistencies regarding the classification systems can lead to inconclusive results in meta-analysis.

The presented results of L&B type I and II fractures strongly support the findings of Polzer et al. and their derived terminology combining type I and type II fractures in ‘epi-metaphyseal’ (L&B type I and II) and metaphyseal (L&B type III) fractures, since they show similar prognosis following operative and nonoperative treatment [9].

Patients with L&B type III fractures presented an overall FAOS score of 93 and the treatment in most cases was operatively. Surgically treated patients tended to have a better functional outcome in comparison to conservative treatment without reaching level of significance. This is in line with other studies recommending operative treatment for these fracture types to the fifth metatarsal bone [14, 16]. In a systematic review Roche et al. concluded, that nonoperative treatment is likely to lead to a higher failure rate than early surgical intervention in L&B type III fractures [14]. In a randomized control trial Mologne et al. demonstrated that screw osteosynthesis lead to a significant lower failure rate in comparison to immobilization in a short leg cast [16]. Due to the different healing progress following operative and nonoperative treatment, Polzer et al. concluded that fractures to the proximal fifth metatarsal bone should be classified as ‘epi-metaphyseal’ and metaphyseal [9]. Looking at the anatomy, this division into two zones follows the watershed line of blood supply of the proximal fifth metatarsal bone. Proximal to this watershed line (L&B type I and II) there is a rich intraosseous blood supply by numerous vessels of the lateral tarsal artery penetrating the non-articular surfaces of the tuberosity. The metaphyseal zone (L&B type III) is supplied by retrograde branches of a discrete nutrient artery [24, 25]. This results in a zone of relative lack of blood supply around this watershed line contributing to a delayed union or nonunion following trauma [9, 24, 25].

In the analyzed study age did not affect the functional outcome, which is in line with previous published studies showing no significant influence of patient age on the functional outcome [12, 13, 26].

Limitations of the study are the retrospective design and small number of patients in certain subgroups. Furthermore, long-term clinical and radiological results were not part of the study and thus the revision rate needs to be interpreted with caution. But to the best of our knowledge, the presented work reports about the largest patient population suffering from fractures to the fifth metatarsal bone using a self-reported patient outcome measurement questionnaire to assess and analyze functional outcome.

## Conclusions

Patients with an L&B type I fracture showed an excellent functional outcome following conservative treatment. The overall FAOS score of L&B type II fractures was satisfactory and conservative management resulted in an equivalent functional outcome in comparison to surgery. Patients with an L&B type III fracture mainly were treated surgically, but difference in FAOS score did not reach level of significance.

## Data Availability

The datasets used and analyzed during the current study are available from the corresponding author on reasonable request.

## Abbreviations

FOAS: Foot and Ankle Outcome Score
L&B: Lawrence and Botte
ORIF: Open reduction and internal fixation
VAS FA: Visual Analogue Scale Foot and Ankle Questionnaire
